# Evolutionary Rate Covariation Across Malaria Parasite Species Enables Inference of Protein Interactions

**DOI:** 10.1093/gbe/evag203

**Published:** 2026-08-17

**Authors:** Helena D Hopson, Radoslaw Igor Omelianczyk, Anayansi Ramirez, Jordan H Little, Nathan Clark, Paul A Sigala, Ellen M Leffler

**Affiliations:** Department of Human Genetics, University of Utah School of Medicine, Salt Lake City, UT, USA; Department of Human Genetics, University of Utah School of Medicine, Salt Lake City, UT, USA; Department of Biochemistry, University of Utah School of Medicine, Salt Lake City, UT, USA; Department of Human Genetics, University of Utah School of Medicine, Salt Lake City, UT, USA; Department of Human Genetics, University of Utah School of Medicine, Salt Lake City, UT, USA; Dept of Biological Sciences, University of Pittsburgh, Pittsburgh, PA, USA; Department of Biochemistry, University of Utah School of Medicine, Salt Lake City, UT, USA; Department of Human Genetics, University of Utah School of Medicine, Salt Lake City, UT, USA

**Keywords:** evolutionary rate, comparative genomics, protein–protein interactions, *Plasmodium*, malaria parasite evolution

## Abstract

Despite publication of the *Plasmodium falciparum* reference genome over 20 years ago, one-third of its genes remain functionally unannotated, and information is limited for many others. Proteins that act in the same pathway or complex tend to experience similar shifts in evolutionary pressure, such that correlated constraints across species can indicate co-functional proteins. To investigate this connection, we calculated relative evolutionary rates across the genome on a phylogeny of 22 *Plasmodium* species and assigned each protein–protein pair a score representing the strength of evolutionary rate covariation (ERC). We show that known pathways and interacting proteins across lifecycle stages in *Plasmodium* have strong ERC signals. By scanning genome-wide for additional proteins showing high ERC with established interacting proteins, we find enrichment of stage expression and physically interacting protein pairs supporting new candidate functions for proteins. More generally, we demonstrate the utility of ERC to prioritize proteins for hypothesis-driven functional follow-up by showing that a protein with little functional characterization (PF3D7_0811600) shows high ERC and co-localizes with high molecular weight rhoptry proteins 2 and 3 (RhopH2 and RhopH3), which form an ion channel that enhances parasite permeability of infected red blood cells. The ERC matrix can be queried to extract *Plasmodium* proteins showing high ERC with any protein of interest to prioritize candidate genes and accelerate discovery of novel functional connections.

Significance A large proportion of proteins in malaria causing parasites (*Plasmodium*) lack functional annotation. Here, we use the genomes of 22 *Plasmodium* species to estimate evolutionary rate covariation (ERC), which identifies functionally related proteins through shared changes in evolutionary constraint. Genome-wide analyses reveal strong ERC signals among known co-functional protein groups and numerous novel interactions. We functionally validate an ERC-predicted interaction by demonstrating co-localization of a candidate protein with a well-characterized complex involved in nutrient uptake, highlighting the utility of this resource for prioritizing proteins for functional follow-up.

## Introduction

*Plasmodium* was responsible for 263 million malaria cases and 597,000 deaths in 2023, the majority of which were caused by *Plasmodium falciparum* (Venkatesan 2025). Despite decades of research, around one-third of *P. falciparum* genes lack functional annotation (Böhme et al. 2019), in part due to limited tools for genetic manipulation and evolutionary distance from common model organisms (de Koning-Ward et al. 2015). Recently, high-throughput mutagenesis screens have been performed in *P. falciparum* (Zhang et al. 2018), the rodent parasite *P. berghei* (Bushell et al. 2017), and the zoonotic monkey parasite *P. knowlesi* (Elsworth et al. 2025; Oberstaller et al. 2025), resulting in gene-level assignments of essentiality and impact on growth. Additionally, a protein–protein interaction network in *Plasmodium* derived from co-migration of protein complexes in native gels queried physical protein–protein interactions among about half of *Plasmodium*'s ∼5,400 proteins (Otto et al. 2014; Hillier et al. 2019). A major goal of *Plasmodium* molecular research is to ascribe more detailed functions to proteins and new functions to uncharacterized genes toward identifying future drug and vaccine targets. Here, we aim to complement existing experimental screens with an evolutionary approach to identify functionally related proteins.

Over the course of evolution, species experience distinct environments and evolutionary pressures. The extent of purifying selection can be quantified as an evolutionary rate, measured as the number of amino acid substitutions per unit time along a branch. A relative evolutionary rate (RER) represents the branch-specific evolutionary rate for a particular protein relative to its average rate of evolution across all branches of a species tree (Kowalczyk et al. 2019; Partha et al. 2019). Additionally, the gene-branch rate is normalized by the average rate on that branch across all genes. In its final form, an RER represents how much faster or slower a protein is evolving in one species relative to all species in the phylogeny. Proteins that have similar functions or act in the same pathway tend to experience similar shifts in evolutionary pressures over time (Goh et al. 2000; Pazos and Valencia 2001; Clark et al. 2012), such that their rates tend to increase (or decrease) on the same branches of the phylogeny. Evolutionary rate covariation (ERC) quantifies the correlation of two proteins’ RERs across a species tree and can thus be used to identify co-functional proteins (Clark et al. 2012; Little et al. 2025). A high ERC value between a pair of proteins can be driven by direct (i.e. physical) or indirect (i.e. shared pathway or function) protein interactions (Clark et al. 2012; Little et al. 2024). This ERC-driven approach to identify co-functional proteins has been applied in a wide range of taxa including mammals (Priedigkeit et al. 2015; Brunette et al. 2019; Kowalczyk et al. 2021), *Drosophila* (Findlay et al. 2014; Raza et al. 2019; Thorpe et al. 2024), and yeast (Clark et al. 2013; Little et al. 2024). Several of these studies have shown experimental validation of ERC-predicted candidates such as connecting new proteins to predefined pathways (Findlay et al. 2014; Brunette et al. 2019; Raza et al. 2019) and localization of an uncharacterized protein (Kowalczyk et al. 2021). Furthermore, in mammals, ERC performed just as well if not better than functional screens (Brunette et al. 2019).

*Plasmodium* species have faced strong and variable evolutionary pressures due to their parasitic lifestyle and frequent host switches, making evolutionary constraint a potentially powerful approach to help predict protein function in these taxa. The *Plasmodium* phylogeny includes parasite species that infect all classes of terrestrial vertebrates and a range of mosquito vectors, with varying degrees of host specificity. The recent increase in the number of reference genomes available for divergent *Plasmodium* species including avian, ape, monkey, and rodent infecting species now make ERC possible in this clade. Here, we calculate ERC for 4,360 proteins and use it to infer new protein functions in *Plasmodium*. We show that there is indeed strong ERC signal among known pathways and co-functional proteins, and that scanning genome-wide reveals clusters of functionally related proteins in networks of high ERC, supported by enrichment in functional datasets and published experimental data. Furthermore, we use ERC to identify a link between a protein with little functional characterization (PF3D7_0811600) and components of a parasite permeability pathway and show that these proteins co-localize. This connection illustrates the utility of ERC as a screening tool to prioritize protein candidates for functional follow-up. We establish ERC as an accessible resource to query proteins and extract high ERC pairs through an Rshiny app (https://lefflerlab.chpc.utah.edu/erc_plasmodium/). Notably, proteins of all 22 *Plasmodium* species included in this study can be queried for ERC interactions. This resource will accelerate the identification of functional roles for uncharacterized proteins and co-functional relationships between proteins, facilitating a more comprehensive understanding of *Plasmodium* biology and discovery of new antimalarial drug and vaccine targets.

## Results

### An ERC Resource for *Plasmodium*

**Fig. 1.**
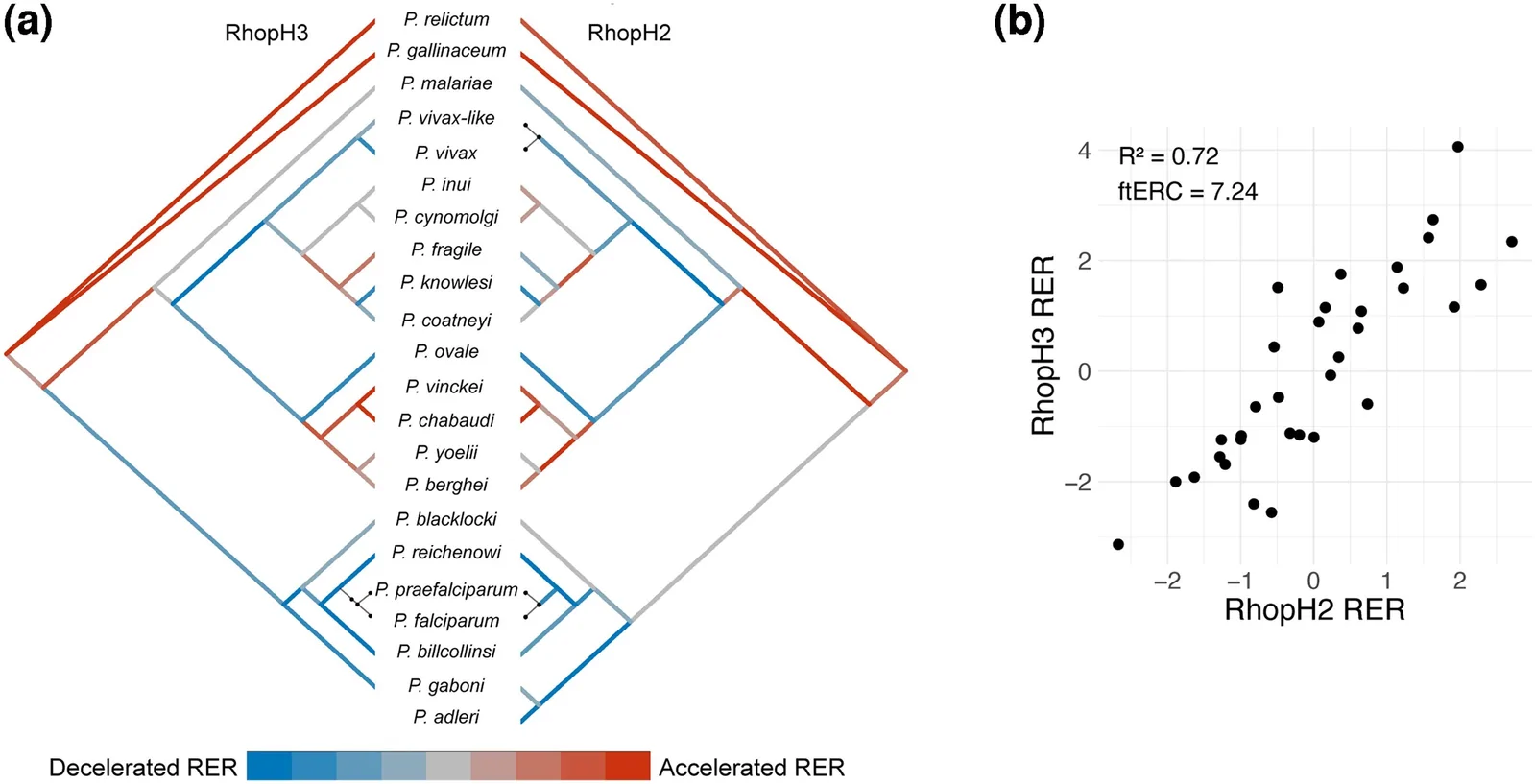
Phylogeny and example of high ERC between RhopH2 and RhopH3. a) The species topology of the 22 *Plasmodium* species used to create the ERC matrix. Dots connect species names to branches. Branches are colored by RER for RhopH3 (left) and RhopH2 (right), with red indicating higher rates (less constraint) and blue indicating lower rates (more constraint). Terminal branches with thin black lines indicate that there were too few substitutions to calculate RER. RhopH3 is missing a terminal branch to *P. gaboni* because there was not a 1:1 ortholog assigned. b) Scatter plot of RER values for RhopH2 and RhopH3 with the Pearson's correlation coefficient (*R*^2^) and Fisher transformed ERC value (ftERC).

To calculate ERC, amino acid sequences of 22 *Plasmodium* species (*P. adleri*, *P. reichenowi*, *P. praefalciparum*, *P. relictum*, *P. vinckei vinckei vinckei*, *P. vivax-like*, *P. vivax*, *P. yoelii yoelii*, *P. ovale curtisi*, *P. malariae*, *P. knowlesi*, *P. inui*, *P. gallinaceum*, *P. gaboni*, *P. fragile*, *P. berghei*, *P. billcollinsi*, *P. blacklocki*, *P. chabaudi chabaudi*, *P. coatneyi*, *P. cynomolgi*, and *P. falciparum*) were downloaded from PlasmoDB (Alvarez-Jarreta et al. 2024) (Table S1). Proteins were assigned to 8,085 ortholog groups using OrthoMCL, and groups with at least 10 1-to-1 orthologs were retained. Following steps used to generate ERC matrices in other taxa (Raza et al. 2019), protein sequences of 1-to-1 orthologs in each group (excluding paralogs) were aligned and RERs were estimated across the phylogeny (Fig. 1a). ERC between each pair of ortholog groups with at least 10 shared species was estimated as the pairwise correlation between their RERs (Fig. 1b). Because 1-to-1 orthology is required for rate estimation, large, duplicated gene families (such as var genes) are absent from this dataset. However, we retain genes with lineage-specific duplications that have sufficient 1-to-1 orthology elsewhere in the phylogeny.

The resulting matrix contains 9,370,728 ERC values across 4,360 proteins. Following Fisher transformation, ERC values ranged from −7.2 to 8.9 with median and mean values both close to 0 (Fig. S1). In total, 4,134 of the ortholog groups (94%) had a single-copy ortholog in *P. falciparum.* Hereafter, we refer to genes by *P. falciparum* gene name and by another representative species for those without single-copy *P. falciparum* orthologs. However, because at least 10 species are represented in each ortholog group, ERC captures proteins that are functionally related across multiple *Plasmodium* species.

ERC is a rank-based approach to prioritize co-functional interactions, such that the higher the ERC value the more likely two proteins functionally interact. Significance is assessed by comparison to the distribution of all ERC values genome-wide. For example, we observed ERC of 7.2, in the 99.9th percentile of all genome-wide ERC values, between RhopH2 and RhopH3, which form an established complex (Fig. 1b) (Cooper et al. 1988; Pasternak et al. 2022). To assess significance of pathways with multiple proteins, we compared the mean of all pairwise ERC values to those of 1,000 random protein sets of the same size.

### Elevated ERC in Known *Plasmodium* Pathways

We selected six pathways that are either well-studied in *Plasmodium* [fatty acid synthesis, hemoglobin digestion, and red blood cell (RBC) invasion] or conserved across eukaryotes and have previously shown strong ERC in other taxa (DNA replication, homologous recombination, and mismatch repair) (Clark et al. 2013; Brunette et al. 2019) (Table S2). We found elevated ERC among proteins involved in five of these pathways (Table 1, empirical *P* < 0.018, Fig. S5) indicating that, as in other taxa, many pathways have a significant ERC signature in *Plasmodium*. We did not observe significantly higher ERC among proteins in the mismatch repair set (Table 1, empirical *P* = 0.5, Fig. S6).

**Table 1:** Mean of all pairwise ERC values between proteins in *Plasmodium* pathways

Pathway	Mean ERC	Empirical *P*-value	Number of genes in ERC matrix
DNA replication	0.37	<0.001	41
Homologous recombination	0.58	0.012	12
Fatty acid synthesis	0.27	0.017	25
Hemoglobin digestion	0.53	0.002	19
Mismatch repair	0.07	0.462	28
RBC invasion	0.95	<0.001	77

To assess ERC signal in other groups of functionally related proteins, we tested for significant mean pairwise ERC among STRING network clusters containing 3 to 500 proteins (Szklarczyk et al. 2023), filtering to keep only proteins with at least one strong confidence interaction (combined score > 700) and in the ERC dataset. Of these clusters, 43.4% (274/631) had significant mean pairwise ERC (empirical *P* < 0.05) (Table S3). In addition, the mean pairwise ERC of 17,174 protein–protein interactions predicted in a *Plasmodium* interactome based on experimental co-migration was significant (0.34, empirical *P* < 0.001) (Hillier et al. 2019). To assess how well ERC captures annotated functional relationships, we tested its ability to predict membership in Biological Process Gene Ontology (GO) terms with at least 10 genes in the ERC matrix (*n* = 117 terms). The median area under the ROC curve (AUC) was 0.67 (Fig. S7a; Table S4), demonstrating that ERC scores are informative of functional group membership. Terms with the highest AUC suggest functional classes particularly amenable to ERC-based screening (Fig. S7b and c). The AUC estimates are likely conservative given that GO annotations are incomplete in *Plasmodium* (Böhme et al. 2019) and only one half of proteins in the ERC matrix had a Biological Process GO annotation. Taken together, these results indicate that many functionally related proteins and pathways in *Plasmodium* show significant ERC. Thus, these proteins and pathways represent good candidates for an ERC-driven approach for prioritization of unannotated functional connections, as subsequently described.

### Genome-wide Scan for Additional Contributors to RBC Invasion

For pathways where components have elevated ERC, the entire ERC dataset can then be searched for additional proteins showing similarly high ERC with proteins in the pathway, as a screen for a new set of candidates that may perform an unrecognized function in the same pathway. We applied this ERC-guided approach to uncover new roles of proteins in red blood cell (RBC) invasion. RBC invasion is essential for parasite growth and both an antimalarial drug and vaccine target, yet many proteins and complex components remain undiscovered (Zainabadi 2016; Counihan et al. 2017; Sherling et al. 2017; Liffner et al. 2021). To this end, we compiled a list of 131 proteins that have been linked to RBC invasion based on functional or localization information from review papers and the malaria parasite metabolic pathway (MPMP) database (Boucher and Bosch 2015; Ginsburg and Abdel-Haleem 2016; Cowman et al. 2017; Liffner et al. 2021) (Table S2). Of the curated RBC invasion protein list, 77 were present in the ERC matrix, spanning multiple stages from pre to post invasion, including surface, microneme, and rhoptry proteins. RBC invasion proteins absent from the ERC matrix include those in multigene families without sufficient 1-to-1 orthology for this approach, such as stevor, SERA, and CLAG genes.

**Fig. 2.**
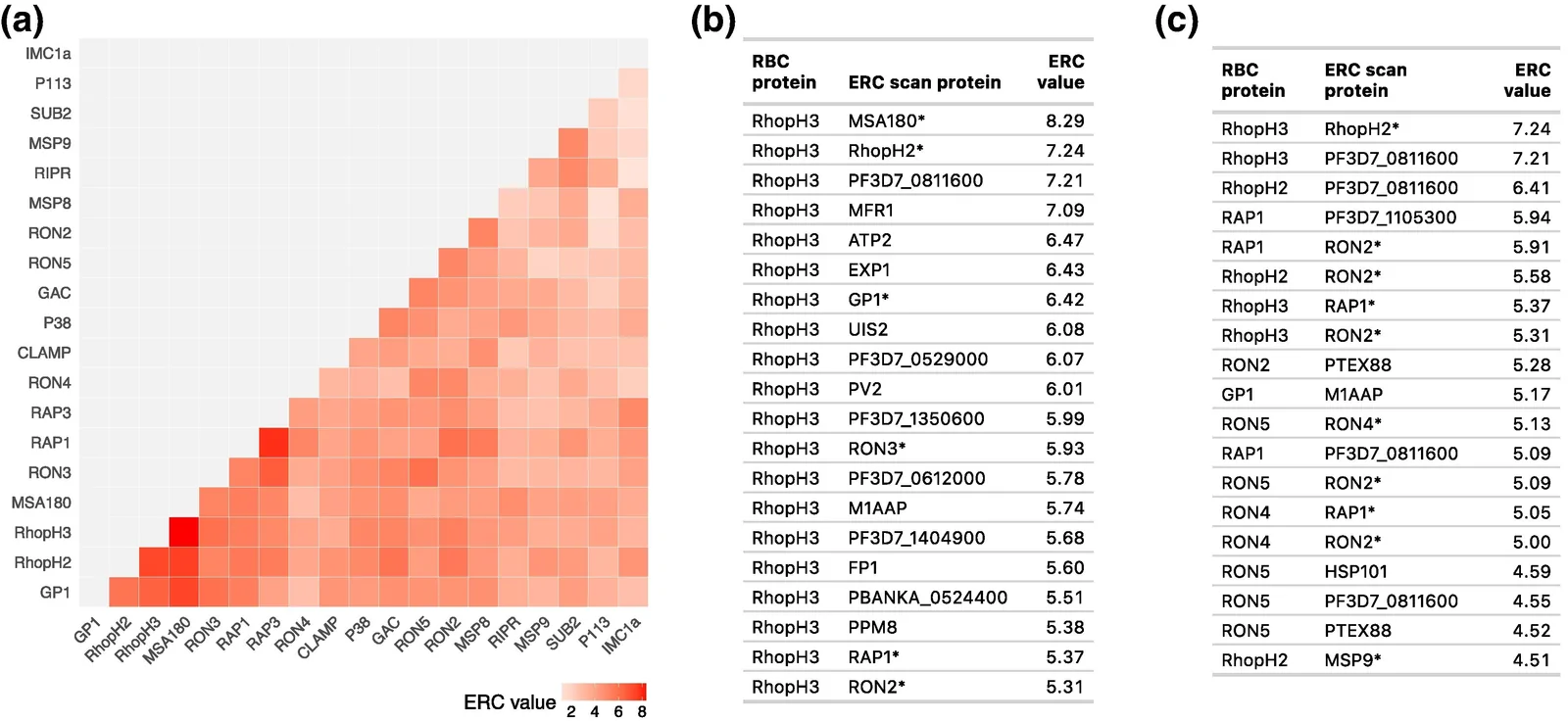
Elevated ERC in RBC invasion proteins. a) Heatmap showing high ERC in a subset of 19 RBC invasion proteins (full heatmap in Fig. S8). b) Table of the 20 proteins showing the highest ERC with RhopH3 genome wide. c) Table of the 19 pairs from the RBC invasion—genome ERC scan that also pair in the interactome from Hillier et al. 2019. Proteins in the curated RBC invasion list are marked with an asterisk. All ERC values in the tables are in the 99.9th percentile of all ERC values genome wide.

The mean pairwise ERC of the 77 RBC invasion proteins was significantly elevated (0.95, empirical *P* < 0.001) (Table 1, Fig. S8). ERC was especially high among a subset of 19 RBC invasion proteins (Fig. 2a). Within this subset, most RBC invasion proteins had their highest genome-wide ERC values with other RBC invasion proteins. For example, RBC invasion protein RhopH3 had six out of its 20 highest ERC values with other RBC invasion proteins in the curated list, including RhopH2 (Fig. 2b).

To scan for proteins with potential functions in RBC invasion, we took proteins with an ERC value in the 99.9th percentile of all values (ERC > 4.48) with any RBC invasion protein, resulting in 550 RBC invasion—genome protein pairs. Of those pairs, 66 contained two RBC invasion proteins (12%) and 484 had one protein not in the RBC invasion list, for a total of 228 unique proteins without a described role in RBC invasion (Table S5). These 228 new candidates for RBC invasion were enriched for expression in asexual blood stages compared to all ERC proteins (Fig. S9). To compare our results with previous scans for RBC invasion proteins, we intersected our candidates with those from a study which used transcriptional similarity to identify a network of 418 candidate RBC invasion proteins (387 after converting to newer gene names and excluding reference genes, 303 in ERC matrix) (Hu et al. 2010). Of our 228 RBC invasion candidates, 39 (17%) were also identified in the Hu et al. co-expression study (Table S6). Only seven of the 26 (27%) reference RBC invasion genes Hu et al. used to identify co-expressed genes were present in the ERC matrix. Scanning for proteins in the 99.9th ERC percentile with these seven reference genes resulted in 98 candidates, of which 20 (20%) were also identified in the co-expression study. Next, we sought to compare our protein pairs with an experimentally derived *Plasmodium* protein–protein interactome (Hillier et al. 2019). Of the 550 RBC invasion—genome scan protein pairs, 368 contained proteins that were both included in the protein–protein interactome study. Of these, 19 pairs also interacted in the interactome study (Fig. 2c). Out of 1,000 permutations of random sets of 368 ERC protein–protein pairs, a number as high as 19 shared protein pairs between ERC and interactome was never observed (empirical *P* < 0.001). This indicates that high ERC with RBC invasion proteins is enriched for physical interactions. Together, these results suggest ERC is identifying both direct (e.g. physical) and indirect (e.g. co-expression) interactions as well as both known and novel interactions, the latter indicating candidate proteins newly implicated in RBC invasion.

### Genome-wide ERC Clustering

**Fig. 3.**
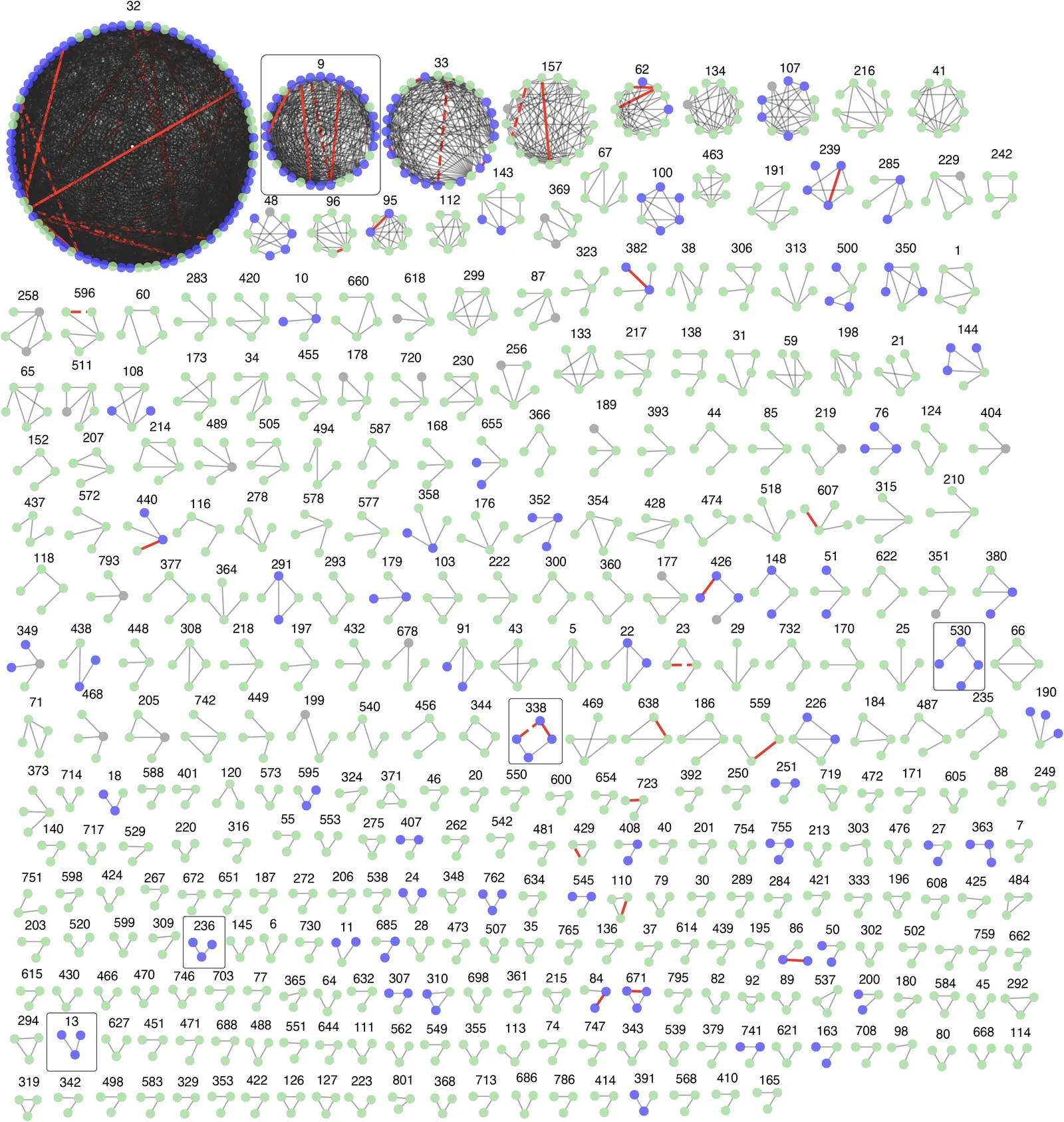
Clustering of the highest ERC genome wide (99.9th percentile ERC > 4.48). Only clusters with at least three proteins with *P. falciparum* orthologs are shown. Gray edges indicate that the ERC value between proteins is above this value. Red edges indicate a confident interaction reported in STRING (combined score >400 and experimental evidence > 0), solid indicates both a STRING interaction and ERC > 4.48 and dashed indicates a confident STRING interaction without an ERC value > 4.48. All confident STRING interactions between proteins with 99.9th percentile ERC values can be found in Table S11. Purple nodes indicate a *P. falciparum* ortholog that is part of an enriched functional term within the cluster (Tables S9 and S10) and gray nodes represent an ortholog group without a *P. falciparum* ortholog. Clusters shown in subsequent figures are boxed.

We identified groups of proteins linked by high ERC by clustering based on the 99.9th percentile of ERC values (>4.48), consisting of 18,742 protein pairs (Table S7). The clustering resulted in 361 clusters with at least three proteins, covering about one-third of ortholog groups in the ERC matrix (1,485/4,360) (Table S8). To link ERC clusters with potential functions, we tested for functional enrichment using STRING database annotations (including GO, KEGG, Reactome, UniProt, and STRING Network Clusters) and GO annotations from PlasmoDB. Of the 326 clusters with at least three proteins with *P. falciparum* orthologs, 61 (∼19%) had at least one significantly enriched functional term (Fig. 3, Tables S9 and S10). Additionally, 83 pairs of proteins in 99.9th percentile of ERC values had a confident STRING interaction (combined score > 400 and experimental evidence score > 0) (Fig. 3, Table S11). These independent links further support the ERC signal between co-functional proteins, yet are limited by small cluster sizes (83% of clusters with <5 proteins) and incomplete annotations in *Plasmodium* (Böhme et al. 2019) (e.g. 9% of proteins in the ERC dataset have no associated GO term) compared to more well-studied taxa.

**Fig. 4.**
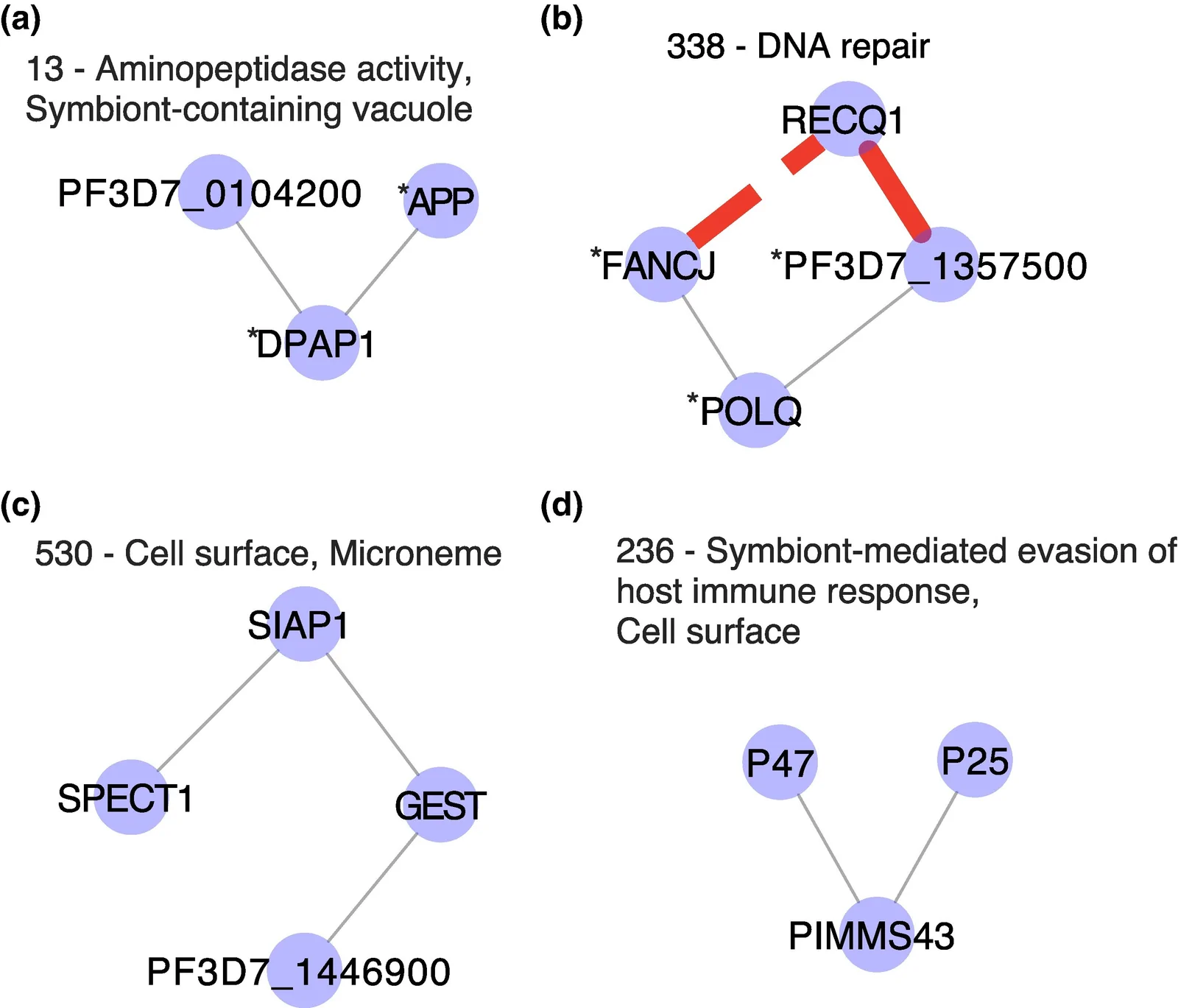
Examples of clusters containing functionally related proteins. Asterisks indicate proteins included in pathways tested in Table 1. Edges and nodes are formatted as described in Fig. 3. a) Cluster 13 contains DPAP1 and APP from the hemoglobin digestion pathway and functional enrichment for GO terms aminopeptidase activity and symbiont-containing vacuole. b) Cluster 338 contains proteins included in DNA replication (RECQ1 and PF3D7_1357500) and homologous recombination (FANCJ) pathways from Table 1. All four proteins are annotated with GO term DNA repair. c) Cluster 530 shows GO enrichment for microneme and cell surface. All four proteins have experimental evidence for functions in sporozoites and transmission from mosquito to liver stages. d) Cluster 236 is enriched for GO terms symbiont-mediated evasion of host immune response and cell surface. All three proteins have experimental evidence for functions in ookinetes and invasion of mosquito midgut.

To assess if the clusters indicate new connections between unannotated proteins and proteins with established functions, we searched for clusters containing proteins within the pathways from Table 1. Cluster 13 includes two proteins in the hemoglobin digestion pathway (Table S2), DPAP1 and APP, along with a third protein, PF3D7_0104200, which is a lipid transporter and does not have a known role in hemoglobin digestion (Fig. 4a). Lipids are thought to be involved in detoxification of by-products from hemoglobin digestion (Matz 2022), and both PF3D7_0104200 and DPAP1 localize within the parasite (Klemba et al. 2004), supporting a potential role in hemoglobin digestion (Fig. 4a). Cluster 338 includes three proteins included in the Table 1 pathways, including DNA replication and homologous recombination (Table S2). All four proteins in the cluster are annotated with the GO term DNA repair (Fig. 4b, Table S9). Three of the four proteins are annotated as “putative”, indicating that their annotations are likely inferred from sequence homology to proteins in other species and have not been experimentally validated. Accordingly, we unable to find any publications on the functional role of these three proteins in *Plasmodium* (FANCJ/PF3D7_1408400/putative FANCJ-like helicase, POLQ/PF3D7_1331100/putative DNA polymerase theta, and PF3D7_1357500/putative DNA helicase). Correlated evolution of these “putative” proteins with experimentally validated *P. falciparum* helicase RECQ1 across the *Plasmodium* phylogeny supports their functional conservation in *Plasmodium* as well.

To assess if ERC-based clustering identified co-functional proteins specific to *Plasmodium* lifecycle stages outside the asexual blood stage, we looked for functionally enriched clusters containing proteins with described roles in mosquito stages. Cluster 530 contains proteins annotated with GO term “cell surface”, including two proteins more specifically annotated with the term “microneme” (SPECT1, GEST) (Fig. 4c, Table S9). The three named proteins localize to the apical end of sporozoites, which mediate transmission from the mosquito to liver. SIAP1 has an early role in sporozoite migration to mosquito salivary glands, and SPECT1 and GEST are involved in later sporozoite traversal to vertebrate host liver cells (Ishino et al. 2004; Engelmann et al. 2009; Talman et al. 2011). The fourth protein in cluster 530 is an unnamed protein (PF3D7_1446900). It was previously identified as a putative glutaminyl-peptide cyclotransferase involved in evasion from the mosquito immune system by posttranslational modification of sporozoite surface proteins (Kolli et al. 2022). Selective pressures from the mosquito immune system could be the driving force of the correlated evolution in this cluster of co-functional proteins. Another example of a cluster of proteins with roles in mosquito stages is cluster 236 (Fig. 4d). All three proteins have reported functions in mosquito immune evasion and are expressed on the surface of the ookinete, which invades mosquito epithelial cells (Dessens et al. 1999; Tomas et al. 2001; Molina-Cruz et al. 2017; Ukegbu et al. 2020). P47 is essential for mosquito transmission and is suggested to underly *Plasmodium* adaptation to new mosquito vector species, as specific combinations of parasite P47 haplotype and mosquito species determine successful infection of the mosquito (Molina-Cruz et al. 2017). High ERC within this cluster raises the possibility that the other proteins (P25, PIMMS43) could be similarly involved in parasite adaptation to mosquitoes. Overall, these results highlight how ERC can identify co-functional proteins across different lifecycle stages and types of pathways, including those that are less accessible experimentally.

### ERC Identifies New Candidates Interacting With RhopH2 and RhopH3

**Fig. 5.**
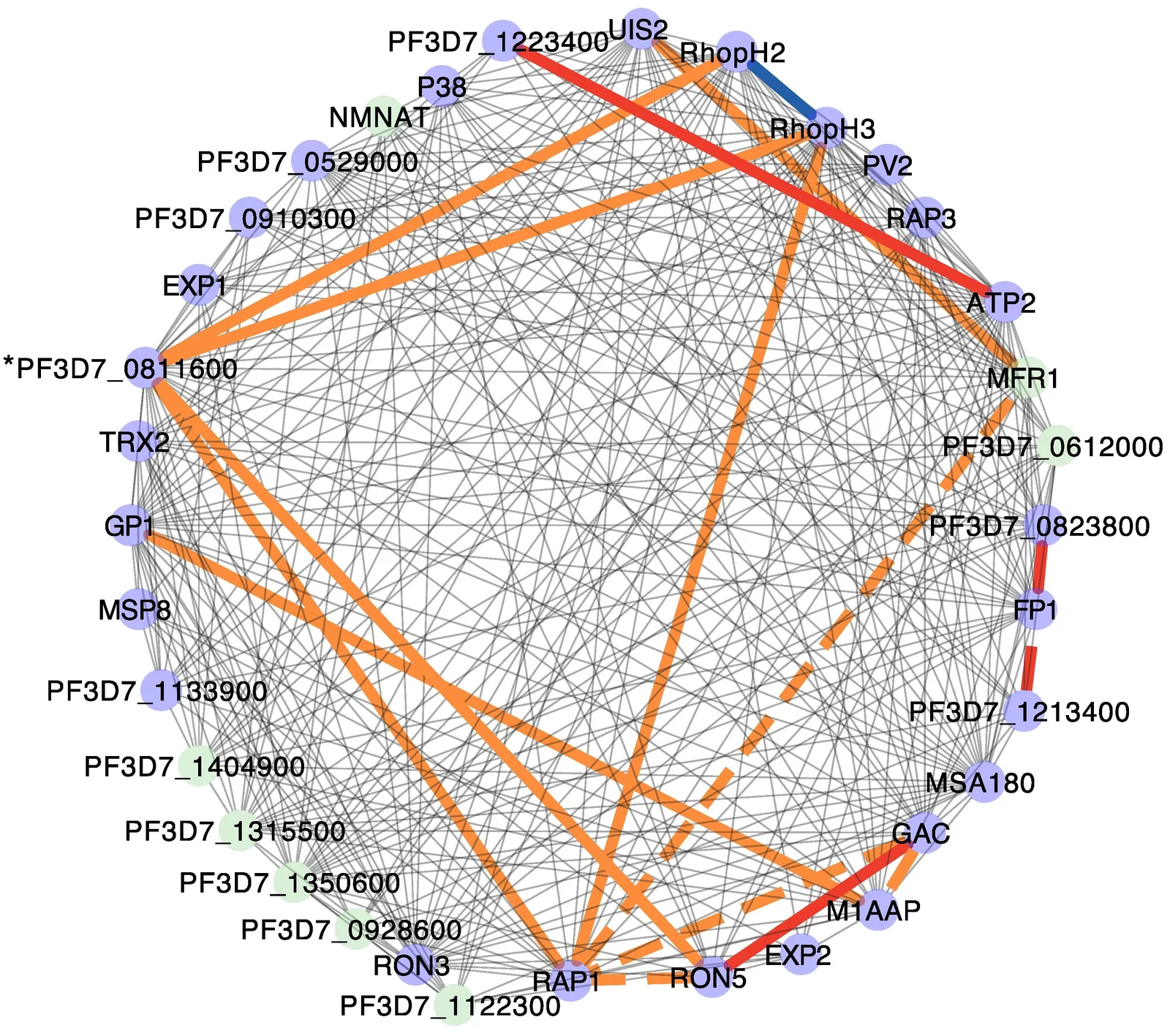
Functional enrichment of cluster 9 containing34 proteins with high ERC. Purple nodes have enriched functional annotations. Red lines indicate STRING interactions as described in Fig. 3. Orange lines indicate protein pairs that also interact in an experimentally derived interactome from Hillier et al 2019. Solid orange lines between seven pairs denote 99.9th percentile of ERC values (> 4.48) and dashed orange lines between four pairs denote less than 99.9 to 95th percentile of ERC values (4.47 to 2.2). The RhopH2 and RhopH3 pair contains all three: 99.9th percentile ERC, interactome, and STRING interactions and is indicated by a blue line. The protein that we followed up experimentally is marked with an asterisk (PF3D7_0811600). The order of nodes differs from Fig. 3 for visualization purposes.

We identified five clusters in which at least one pair of proteins with high ERC were also found to interact in an experimentally derived interactome (Hillier et al. 2019), for a total of 20 protein pairs (Table S12). Cluster 9 contains 12 such pairs and is enriched for GO terms including apical complex and rhoptry, which are the structures in the RBC-invading merozoite that facilitate initial binding and later discharge their contents into the host RBC during invasion (Liffner et al. 2021). This cluster includes six known rhoptry proteins: RAP1, RAP3, RON3, RON5, RhopH2, and RhopH3. RhopH2 and RhopH3 are part of the RhopH complex, a key component of the *Plasmodium* surface anion channel (PSAC) that is inserted on the infected host RBC membrane to facilitate nutrient uptake (Nguitragool et al. 2011; Counihan et al. 2017; Ito et al. 2017). The scope of PSAC affiliated proteins is not known (Ito et al. 2017; Counihan et al. 2021), and this cluster suggests new proteins that could be involved. We focused on PF3D7_0811600, which had high ERC and previous interactome-predicted interactions with RhopH2, RhopH3, and RAP1 (Hillier et al. 2019) (Fig. 5).

**Fig. 6.**
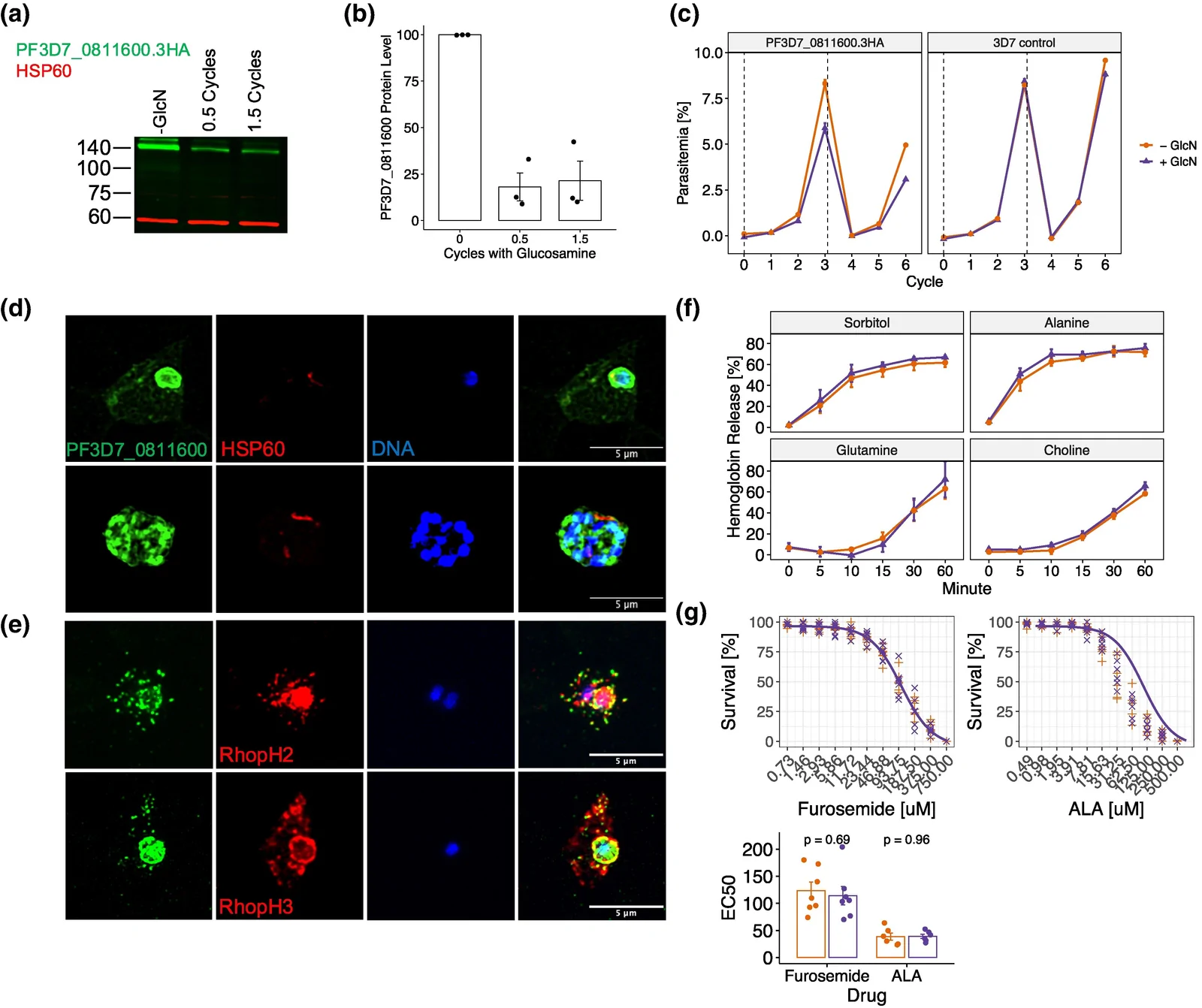
PFD7_0811600 co-localizes with PSAC but does not affect solute import. a) Western blot of PF3D7_0811600 shows protein knock-down after addition of glucosamine (GlcN) for 0.5 and 1.5 cycles. HSP60 is a marker for the mitochondrion and shown as a control. b) Quantification of PF3D7_0811600 protein level after knock-down. c) Growth of parasites with (+GlcN) and without (−GlcN) knock-down of PF3D7_0811600 and in 3D7 parasites without tagged PF3D7_0811600 (right). d) Localization of PF3D7_0811600 in infected red blood cells in early-stage parasites (top row) and late-stage parasites (bottom row). e) Co-localization of PF3D7_0811600 with RhopH2 (top row) and RhopH3 (bottom row). f) Cell lysis resulting from treatment with solutes, with and without PF3D7_0811600 knock-down. g) Survival with furosemide and 5-aminolevulinic acid treatment with and without PF3D7_0811600 knock-down.

To functionally validate the predicted interactions between PFD7_0811600 and RhopH2/RhopH3 suggested by high ERC, we obtained a *P. falciparum* line with an endogenously modified PF3D7_0811600 gene to encode a triple hemagglutinin (3xHA) tag and a glmS ribozyme sequence in the 3′ untranslated region, allowing inducible knock-down of gene expression upon addition of glucosamine (Paoletta et al. 2025). Addition of glucosamine to tightly synchronized ring-stage parasites reduced protein levels by 80% within the same cycle and remained constant over multiple life cycles (Fig. 6a and b). Parasites showed only a minor growth defect, despite the protein being predicted to be essential in *P. falciparum* and *P. knowlesi* (Fig. 6c) (Zhang et al. 2018; Oberstaller et al. 2025). We used immunofluorescence to test for subcellular localization of PFD7_0811600 in fixed parasites and saw an accumulation of the protein at the parasite periphery and in a punctate pattern in the infected RBC in early-stage parasites (Fig. 6d). In late-stage parasites, the protein appeared to localize to distinct foci in the forming merozoite. The protein co-localized with both RhopH2 and RhopH3 (Pearson coefficient of 0.6 ± 0.0833 and 0.541 ± 0.119, respectively), with a very high M1 value (0.93 ± 0.0737 with RhopH2 and 0.898 ± 0.137 with RhopH3), and a lower M2 value (0.704 ± 0.159 with RhopH2 and 0.626 ± 0.171 with RhopH3; Fig. 6e and Fig. S10a). This pattern suggests that PF3D7_0811600 almost entirely localizes proximal to PSAC, but also that there is a subset of PSAC without PF3D7_0811600. We were unable to detect enrichment of RhopH3 in co-immunoprecipitations followed by western blot using PF3D7_0811600 as bait (Fig. S10b). This observation, together with the skewed M1 and M2 values, suggests a transient interaction between PF3D7_0811600 and the PSAC.

To test for the role of PF3D7_0811600 in PSAC function, we measured solute import into the infected RBC upon protein knock-down. Import of sugars such as sorbitol or amino acids such as alanine require PSAC activity and result in lysis of the cell at high concentrations (Wagner et al. 2003). Indeed, knock-down of RhopH2 has previously been shown to reduce the uptake of sorbitol and alanine (Counihan et al. 2017). However, we observed no difference in cell lysis upon PF3D7_0811600 knock-down, quantified as percent of hemoglobin release, after treatment with sorbitol, choline, alanine or glutamine (Fig. 6f). This observation mirrors a recent publication where knock-down of none of 13 putative RhopH2 interacting proteins changed sorbitol uptake (Jonsdottir et al. 2021). We further tested whether knock-down of PFD7_0811600 changes parasite susceptibility to treatment with the PSAC inhibitor furosemide (Kirk et al. 1994) or 5-aminolevulinic acid, which is imported through the PSAC (Sigala et al. 2015), but we did not detect a shift in EC_50_ for either drug (Fig. 6g). This observation, together with a lack of change in solute import, suggests that PFD7_0811600 is not directly involved in or required for PSAC function, consistent also with the lack of in vitro growth phenotype upon knock-down. Alternatively, as we only achieved partial knock-down with 20% of the protein remaining within the cell (Fig. 6a and b), these low amounts of protein may be sufficient to facilitate its function.

## Discussion

We have generated a pairwise ERC dataset for 4,360 *Plasmodium* proteins calculated across 22 *Plasmodium* species. We found that diverse groups of co-functional proteins and pathways across multiple lifecycle stages show signatures of high ERC, demonstrating this evolutionary-based approach to be a useful screening and/or discovery tool for *Plasmodium* protein function, as in other eukaryotic taxa (Clark et al. 2013; Findlay et al. 2014; Priedigkeit et al. 2015; Brunette et al. 2019; Raza et al. 2019; Kowalczyk et al. 2021; Thorpe et al. 2024). We illustrated this utility of ERC as a screening tool by two approaches. First, we scanned genome-wide for proteins showing high ERC with co-functional proteins involved in RBC invasion to identify a set of new candidates, which are enriched for blood stage expression and physical interactions. Second, by clustering the highest ERC values genome-wide, we identified uncharacterized proteins that cluster with established co-functional proteins. From this, we confirmed co-localization of an ERC-predicted interaction between PSAC components RhopH2/H3 and a recently characterized protein, PF3D7_0811600.

Recent work found PF3D7_0811600 localized to the parasitophorous vacuole membrane (PVM), which separates the parasite from the host cell cytosol, and knock-down of PF3D7_0811600 led to reduced CD36-mediated cytoadhesion (Paoletta et al. 2025). Similarly, the *P. berghei* ortholog of PF3D7_0811600, called merozoite attachment protein 1 (MAP1), also localized to the PVM and loss resulted in reduced sequestration. PF3D7_0811600 is also referred to as PfMAP1 and is annotated as “protein MAP1, putative” in PlasmoDB. Here, we use gene names from PlasmoDB release 54 annotations where it is unnamed. The co-localization of some but not all PSAC components with PF3D7_0811600 suggests a transient or heterogeneous interaction between these proteins. For example, PF3D7_0811600 could be involved in trafficking of PSAC to the host RBC membrane and dissociate after PSAC installation on the RBC surface. To reach the host RBC membrane, RhopH2 and RhopH3 pass through the *Plasmodium* translocon of exported proteins (PTEX) (Pasternak et al. 2022), which is a protein complex that transports proteins across the PVM and into the host cytosol (de Koning-Ward et al. 2009). In two previous studies, PF3D7_0811600 was among the proteins that immunoprecipitated with a PTEX intermediate complex, suggesting it could interact with the PTEX components (Mesén-Ramírez et al. 2016; Miyazaki et al. 2021). Twelve of the other 29 immunoprecipitated proteins identified in Miyazaki et al. (Miyazaki et al. 2021) had ERC values in the 99th percentile (ERC > 3.2) with PF3D7_0811600 (Tables S13 and S14). These proteins included both RhopH2 and RhopH3, HSP101 and EXP2 (two of three core PTEX components) (Garten et al. 2018), RON3 which is essential for PTEX protein transport function (Low et al. 2019), EXP1 which is required for correct localization of core PTEX component EXP2 (Nessel et al. 2020), and PTEX associated protein P113. Seven of the 19 immunoprecipitated proteins identified in Mesén-Ramírez et al. (2016) had ERC in the 99th percentile with PF3D7_0811600, again including HSP101 and EXP2, and PTEX88, an accessory PTEX component (Table S15). Interestingly, neither study pulled out the second accessory PTEX component, TRX2, yet we observe strong ERC between TRX2 and other PTEX components. However, we do not see strong ERC between core PTEX protein PTEX150 and the other four PTEX proteins, despite its function as a physical adapter linking HSP101 and EXP2 (Ho et al. 2018) (Fig. S11). Further, GO enrichment of proteins in the 99th percentile of ERC with PF3D7_0811600 (Table S13) revealed significant terms including PTEX complex, Maurer's cleft, channel activity, transmembrane transporter activity, and host cell cytoplasm/membrane/surface binding (Fig. S12, Tables S16 and S17). While we did not see an effect of PF3D7_0811600 on transport of solutes tested in this study (sorbitol, choline, alanine, glutamine, and 5-aminolevulinic acid), it is possible that PF3D7_0811600 is involved in transport of other solutes, as the GO enrichment for transporter functions suggests, or that sufficient activity remained even after knocked down and a full knockout would be required to observe an effect on transport. Alternatively, PF3D7_0811600 could be more generally involved in global export of proteins as high ERC with PTEX components and immunoprecipitation with intermediate PTEX complexes suggest. Additionally, ERC signal could be driven in part by the parasite sexual stages where PF3D7_0811600 has been shown to localize to vesicles involved in gametocyte egress, along with several other proteins in the ERC cluster (Sassmannshausen et al. 2024).

High ERC can reflect both direct and indirect protein–protein interactions. Here, we observed high ERC between direct, physically binding protein–protein interactions, for example: p12 and p41 (3.7; *P* = 0.005) (Taechalertpaisarn et al. 2012) and the RON complex consisting of RON2, RON4, and RON5 (5.1; *P* = 0.001) (Riglar et al. 2011; Tonkin et al. 2011; Cowman et al. 2017). We also saw high ERC between indirect protein interactions such as proteins within the same pathway (Table 1) and co-functional proteins (Figs. 4 and 5). Previous work in yeast showed that ERC was stronger between indirect protein interactions compared to direct interactions, suggesting nonphysical forces such as changes in evolutionary constraint, expression, codon adaptation, or essentiality are primary drivers of ERC (Little et al. 2024).

The high ERC observed between RhopH2 and RhopH3, driven by correlated RERs across the species tree, appears to be loosely correlated with host species. The RhopH2/H3 proteins had accelerated RERs in rodent (*P. berghe*i, *P. vinckei*, *P. chabaudi*, *P. yoelii*) and avian malaria parasites (*P. relictum*, *P. gallinaceum*) and decelerated RERs in the ape-infecting (*P. ovale*, *P. malariae*, and Laverania clade containing *P. falciparum*) and monkey-infecting species (*P. knowlesi*, and *P. vivax* and *P. vivax-like*) (Fig. 1a). The core structure of the PSAC, including the RhopH2/H3 complex, is thought to be conserved across *Plasmodium* species (Counihan et al. 2021), but differences in affinities of PSAC inhibitors between *Plasmodium* species suggest it may serve different functions (Lisk and Desai Sanjay 2005; Trickey et al. 2024). It is plausible that shifts in RERs of RhopH2/H3, and other highly correlated transport proteins (Fig. 2b and 5), could be due to different nutrient availability or requirements imparted by unique host species environments, resulting in alteration of nutrient acquisition mechanisms. Indeed, parasite species differ in sensitivity to drugs targeting metabolic pathways (Chan et al. 2013), essentiality of transporters (Hart et al. 2016) and metabolic pathways (van Schaijk Ben et al. 2014), and complete loss of metabolic pathways (Déchamps et al. 2010).

While we have shown that ERC contains information about functional relationships and can be used as a screening method, it is important to note that not all functional interactions show an ERC signal. For instance, many STRING network clusters did not have significantly elevated ERC. Many explanations are possible for lack of ERC including pleiotropy, peripheral membership in a cluster, and moonlighting proteins with multiple distinct functions resulting in different evolutionary pressures. We found the GO term “membrane” enriched among genes with the strongest ERC values (top 25% of genes with the most edges in the ERC network), suggesting membrane proteins may tend to experience shared strong evolutionary pressures.

We did not observe elevated ERC for the mismatch repair pathway (Table 1). Although mismatch repair proteins are generally conserved across eukaryotes and show elevated ERC in yeast and mammals (Clark et al. 2013; Groothuizen and Sixma 2016), the mismatch repair pathway appears to be less conserved in *Plasmodium* (Bethke et al. 2007; Shankar and Tuteja 2008; Ahmad and Tuteja 2014; Siao et al. 2020) and may also be under selection related to drug treatment (Castellini et al. 2011). It is possible that our mismatch repair protein list does not show elevated ERC because it contains proteins that serve other functions in *Plasmodium*. Within our mismatch repair protein list, there are subsets of proteins that have high ERC. For example, a group of putative exonucleases (PF3D7_0204600, PF3D7_1362500, PF3D7_0105900, and PF3D7_0204800) and the DNA helicase UvrD (PF3D7_0514100) cluster together with higher ERC values (Fig. S6). Additionally, replication proteins (RPA1; PF3D7_0409600, PF3D7_0904800 and RPA3; PF3D7_1442100) cluster together. Some proteins in the heatmap show little ERC with others, including two putative mismatch repair proteins (PMS1; PF3D7_0726300, PF3D7_0505500; MSH6). Proteins within the top 100 ERC values with these two putative mismatch repair proteins were enriched for GO terms related to RNA metabolism and processing. High ERC with PMS1 included three U3 small nucleolar RNA associated putative proteins (UTP7; PF3D7_0722600, UTP12; PF3D7_1448000, UTP4; PF3D7_1333600) which are part of the small subunit processome for ribosome biogenesis (Table S18). These results indicate potential divergent functions of putative mismatch repair proteins in *Plasmodium*. Since ERC is estimated using *Plasmodium* sequences, it is a *Plasmodium*-specific inference of protein function and may be particularly useful to predict the function of divergent proteins in *Plasmodium*.

## Materials and Methods

### *Plasmodium* Reference Genomes

Amino acid FASTA files (ending in “_AnnotatedProteins.fasta”) were downloaded from PlasmoDB release 50 (https://plasmodb.org/plasmo/app/downloads) for each of 22 *Plasmodium* species: *P. adleri* G01, *P. reichenowi* CDC, *P. praefalciparum* G01, *P. relictum* SGS1, *P. vinckei vinckei vinckei*, *P. vivax-like* Pvl01, *P. vivax* P01, *P. yoelii yoelii* 17X, *P. ovale curtisi* GH01, *P. malariae* UG01, *P. knowlesi* H, *P. inui* SanAntonio1, *P. gallinaceum* 8A, *P. gaboni* G01, *P. fragile* Nilgiri, *P. berghei* ANKA, *P. billcollinsi* G01, *P. blacklocki* G01, *P. chabaudi chabaudi*, *P. coatneyi* Hackeri, *P. cynomolgi* M, and *P. falciparum* 3D7 (Table S1). We used the “PlasmoDB-54_Pfalciparum3D7.gff” file to annotate protein names and descriptions also downloaded from the link above.

### Ortholog Assignment and Alignment

Ortholog groups within the 22 species were assigned using OrthoMCL v2.0.91 (Li et al. 2003). Protein sequences flagged as short or highly repetitive by OrthoMCL were excluded. For proteins with multiple isoforms, a single isoform was kept (selecting the isoform annotated with “.1'). If multiple genes from the same species were present in an ortholog group, all genes from that species were removed, and only 1:1 orthologs from other species were retained. Furthermore, only ortholog groups with at least ten 1:1 orthologs were kept. We refer to ortholog groups by the *P. falciparum* gene name when one is present. If an ortholog group did not contain a single-copy *P. falciparum* ortholog, it was named by the *P. berghei* ortholog, followed by *P. vivax*, *P. reichenowi*, *P. coatneyi*, *P. malariae*, *P. ovale*, *P. knowlesi*, *P. cynomolgi*, *P. inui*, *P. fragile*, *P. chabaudi*, *P. yoelii*, *P. vinckei*, *P. gaboni*, *P. adleri*, *P. blacklocki*, *P. billcollinsi*, *P. praefalciparum*, *P. vivax-like*, *P. relictum*, and *P. gallinaceum* ortholog. Protein sequences of each one-to-one ortholog group were aligned using MUSCLE v3.8.31 (Edgar 2004).

### RER and ERC Estimates

The species topology was inferred using published phylogenetic trees available for subsets of species as well as literature consensus (Rutledge et al. 2017; Böhme et al. 2018; Otto et al. 2018; Abu-Elmakarem et al. 2024) (Fig. 1a). This species topology and the multiple sequence alignment of each ortholog group were used to estimate individual maximum likelihood gene trees with the estimatePhangornTreeAll wrapper within the R package RERconverge (v0.1.0) (Schliep 2011; Kowalczyk et al. 2019), with default options and the LG amino acid substitution matrix (Le and Gascuel 2008). Protein relative evolutionary rates (RERs) were estimated by normalizing a gene's branch length in each species to its average evolutionary rate across the species tree and to the average rate of all other genes on that branch, detailed elsewhere (Chikina et al. 2016; Partha et al. 2017; Kowalczyk et al. 2019; Partha et al. 2019). For a random set of 50 genes, we also aligned sequences with PRANK v.170427 (Löytynoja 2014) and verified a high correlation (mean *R*^2^ = 0.99) between branch lengths, indicating robustness to alignment algorithm. ERC was calculated as the Pearson correlation of the RERs between two ortholog groups across the phylogeny, followed by a Fisher transformation to normalize for comparisons between ortholog groups with different numbers of branches, as previously described (Raza et al. 2019; Little et al. 2024; Little et al. 2025). ERC was calculated between all pairs of ortholog groups with at least 10 shared species. The resulting genome-wide matrix of protein–protein pairwise ERC values was used in all downstream analyses and can be found under data availability.

### ERC Analysis of Proteins in Known Pathways

Lists of proteins with established roles in pathways were obtained from MPMP databases (Ginsburg and Abdel-Haleem 2016) (Table S2). Specifically, under “maps” and “map details”, we extracted the gene names under fatty acid synthesis II (https://mpmp.huji.ac.il/maps/facidsynthesispath.html), DNA replication (https://mpmp.huji.ac.il/maps/dnareplication.html), double strand break repair and homologous recombination (https://mpmp.huji.ac.il/maps/dsbRecomb.html), hemoglobin digestion (https://mpmp.huji.ac.il/maps/hemoglobinpolpath.html), and DNA mismatch repair (https://mpmp.huji.ac.il/maps/DNArepair.html) pathways. The RBC invasion gene set was compiled from the MPMP website (https://mpmp.huji.ac.il/maps/Merozoiteproteins.html) and several review papers (Boucher and Bosch 2015; Cowman et al. 2017; Liffner et al. 2021). This set includes both proteins with both known functions in RBC invasion and those with putative roles due to their localization to rhoptries, micronemes, or the merozoite surface (Table S2). To assess the strength of ERC for each pathway, we calculated the mean ERC across all pairs of pathway proteins. We then randomly sampled protein sets of the same size and computed the pairwise mean ERC in the same way, repeating 1,000 times to determine an empirical *P*-value. This same process was repeated for *P. falciparum* STRING network clusters downloaded from https://string-db.org/ (version 12.0). Proteins in STRING network clusters were retained if they were in the ERC dataset and had at least one high confidence interaction (combined score > 700) with another protein in the cluster.

### Comparison With an Experimental Interactome

We downloaded a *Plasmodium* protein interactome derived from experimental protein co-migration combined with other evidence for interaction including co-expression, interacting domains, co-evolution, and phenotype data (Hillier et al. 2019). To assess the overlap between high ERC values and interactions identified in the interactome, we only considered protein pairs that were included in both datasets. A majority (2,226/2,672, 83%) of the *P. falciparum* proteins queried in the interactome dataset were also in the ERC matrix. About half (2,226/4,134, 53%) of the *P. falciparum* proteins in the ERC matrix were also queried in the interactome dataset. The mean ERC value of the 17,174 *P. falciparum* protein interactions from the interactome dataset was calculated and an empirical *P*-value determined similarly as for pathways, except that proteins were randomly sampled only from those that were also in the interactome.

### Prediction of GO Term Membership From ERC With glmnet

We fit elastic net logistic regression models using the glmnet R package (v5.0) (Tay et al. 2023) for each GO Biological Process term with at least 10 annotated members in the ERC matrix, using GO annotations from PlasmoDB (release 54). For each GO term, a binary vector was constructed across all *P. falciparum* proteins in the ERC matrix (1 = annotated member, 0 = nonmember). The features were each protein's ERC values with all other proteins in the matrix. Models were fit with cv.glmnet(), using an elastic net penalty (alpha = 0.5) and 10-fold cross-validation (nfolds = 10), optimizing the ROC-AUC (type.measure = “auc”). The AUC at lambda.min was reported for each term. Non-*P. falciparum* proteins were excluded from the matrix prior to modeling.

### Analysis of Top ERC Values Genome-Wide and With RBC Invasion Proteins

We selected the 99.9th percentile of ERC values (>4.48) across the entire dataset to explore genome-wide patterns of the strongest ERC values. To identify groups of potential co-functional proteins among the top genome-wide ERC values, we applied Markov chain clustering followed by Leiden clustering to the ERC pairs using clusterMaker2 and visualized the clusters within cytoscape (v3.10.1) (Shannon et al. 2003). For functional enrichment analyses, we used clusterProfiler (v4.12.6) with *P. falciparum* Gene Ontology annotations downloaded from PlasmoDB (release 54) (Wu et al. 2021). We also used the STRING annotation databases and the get_enrichment function in the STRINGdb R package (v2.16.4) for functional enrichment. We used all *P. falciparum* orthologs in the ERC matrix as the background.

We also selected the 99.9th percentile of ERC values with known RBC invasion proteins to evaluate for functional candidates and co-functional relationships in the process of RBC invasion. We tested for an enrichment of pairs also in the interactome pairs from Hillier et al. 2019 by randomly sampling sets of the same number of ERC pairs from the interactome filtered ERC dataset described above. To test for enrichment of expression of RBC invasion ERC candidates in blood stages, we used *P. falciparum* single cell gene expression data from the Malaria Cell Atlas covering the asexual blood stages (rings, schizonts, and trophozoites) (Howick et al. 2019). We considered a gene expressed in a particular stage if it was expressed in at least 25% of cells from that stage. To intersect our RBC invasion candidates with previously identified candidates based on transcriptional patterns, we downloaded Table S6 from Hu et al. (2010) and converted older gene identifiers using PlasmoDB release 54 gene aliases. The RBC invasion genes used as the well-established reference genes in their paper were excluded prior to intersection with our candidates.

### Parasite Culture, Transfection, Growth Assays, and Drug Assays

Parasite culture, transfection, and selection were performed as previously described (Trager and Jensen 1976; Fidock and Wellems 1997). Parasites were cultured in RPMI1640 supplemented with 2.5 g/L Albumax I Lipid-Rich BSA, 15 mg/L hypoxanthine, 110 mg/L sodium pyruvate, 1.19 g/L HEPES, 2.52 g/L sodium bicarbonate, 2 g/L glucose, and 10 mg/L gentamicin. Cultures were maintained at 2.5% hematocrit in human erythrocytes obtained from the University of Utah Hospital blood bank, at 37 °C, and 5% CO_2_. Parasites were kept synchronous through treatment with 5% (w/v) Sorbitol. Parasite growth and viability was routinely controlled via Giemsa stained thin blood smear. Knock-down of PF3D7_0811600 was initiated by addition of 2.5 mM glucosamine (Prommana et al. 2013). Late-stage PF3D7_0811600.3HA.glms parasites were enriched using MACS magnetic columns (Ribaut et al. 2008). Continuous growth assays and drug assays were performed as previously described (Okada et al. 2022). Parasites treated with ALA were subjected to two exposures to broad-wavelength white light (2 min each, 24 and 36 h after addition of ALA) on an overhead projector (Sigala et al. 2015).

### Western Blot Analysis

Late-stage parasites were lysed in PBS with 1% Triton X-100, 0.1% sodium dodecyl sulfate, 1:1,000 Pierce Universal Nuclease for Cell Lysis and 1:200 SelleckChem Protease Inhibitor Cocktail (EDTA free, 100× in DMSO) for 30 min at 4 C. Lysates were clarified through centrifugation at 13,000 × *g* for 30 min at 4 C. Clarified lysates were mixed with loading dye loaded onto 10% polyacrylamide gels and fractionated via SDS-PAGE. Proteins were transferred onto nitrocellulose membrane. Membranes were airdried, rehydrated in PBS and then blocked in PBS with 5% skimmed milk powder for 30 min at room temperature. Membranes were incubated with primary antibodies diluted in PBS + 1% skimmed milk +0.1% Tween20 overnight at 4 C with gentle agitation. Membranes were washed thrice with PBS + 0.1% Tween20 and incubated with secondary antibodies for 1 h at room temperature. Secondary antibodies were diluted in PBS + 1% skimmed milk + 0.1% Tween20. Membranes were washed again thrice with PBS + 0.1% Tween20, rinsed with PBS and airdried. Membranes were scanned on a Li-Cor Odyssey CLx. Band intensities were measured using Li-Cor Image Studio Version 5.2. To preclear primary antibody solutions containing antibodies against RhopH2 or RhopH3 (Ito et al. 2017), samples of uRBCs were prepared and processed as described above. Primary antibody solutions were incubated with membranes loaded with uRBC samples for 1 h at room temperature. Precleared primary antibody solutions were then transferred onto membranes with the samples of interest.

### Immunofluorescence Analysis

All steps were performed at room temperature. Parasite cultures were washed once using PBS and diluted to 0.1% hematocrit in PBS. Parasite suspension was transferred to poly-D-lysine hydrobromide (molecular weight 30,000 to 70,000) coated cover slips and left to settle for 30 min. Coverslips were rinsed with PBS and cells were fixed with 4% paraformaldehyde in PBS for 30 min. Fixed cells were permeabilized with PBS + 0.1% Triton-X100 for 5 min. Cells were blocked with PBS + 3% BSA for 30 min, followed by incubation with primary antibodies in PBS + 1% BSA for 1 h. Cover slips were washed twice with PBS for 5 min. Cells were finally incubated with secondary antibodies and Hoechst33342 (1 µg/mL) in PBS + 1% BSA for 1 h. After two more washes in PBS for 5 min, cells were sequentially dehydrated in 70%, 90%, and 100% ethanol for 3 min each. Cover slips were left to airdry and mounted onto glass slides using Invitrogen ProLong Diamond Antifade Mountant with DAPI. Cells were imaged within the next 7 d on a Nikon ECLIPSE Ti2-E inverted microscope with a Yokogawa CSU-W1 Confocal Scanner Unit (with 50 µm pinhole disk), a Gataca Systems Live-SR Super Resolution Module and an Apo TIRF 100× Oil DIC N2 objective using a Teledyne Kinetix22 Back Thinned sCMOS Camera (6.5 µm pixel size and 2400 × 2400 sensor). Images were taken using the Nikon NIS elements AR/HC software package (versions 5.42 or 6.02) and deconvoluted using NIS elements Batch Deconvolution (version 5.30.01) in automatic mode. Images were analyzed using FIJI (Schindelin et al. 2012) (Version 2.16.00, Java 1.8.0_322) and co-localization was calculated using the plugin JaCOP (Bolte and Cordelières 2006) (Version 2.1.4).

### Co-immunoprecipitation

To detect direct protein–protein interactions between PF3D7_0811600 and RhopH2 or RhopH3, late-stage parasites were harvested using MACS magnetic columns (Ribaut et al. 2008). Parasites were lysed in PBS with 1% Triton X-100, 1:1,000 Pierce Universal Nuclease for Cell Lysis and 1:200 SelleckChem Protease Inhibitor Cocktail (EDTA free, 100× in DMSO) for 30 min at 4 C. Lysates were clarified through centrifugation at 13,000 × *g* for 30 min at 4 C. Clarified lysates were mixed with Pierce Anti-HA Magnetic Beads at a 1:10 ratio (1 volume bead slurry:10 volumes infected RBC pellet) and incubated at 4 C overnight with gentle agitation. Beads were washed 5 times with ice cold PBS with 1% Triton X-100 for 5 min at 4 C with gentle agitation. Bound proteins were eluted in nonreducing loading dye for 5 min at 95 C. Samples were supplemented with 2-mercaptoethanol to a final concentration of 3% (v/v) and incubated at 95 C for 5 min. Western blotting was performed as described above.

### Hemoglobin Release Assay

The hemoglobin release assay to assess PSAC function was performed as previously described (Kirk et al. 1994). In brief, late-stage PF3D7_0811600.3HA.glms parasites (untreated or treated with glucosamine for 48 h) were harvested using MACS magnetic columns (Ribaut et al. 2008). 1/7th of the parasite pellet was incubated in PBS with 1% Triton X-100 for 15 min at room temperature to achieve 100% hemoglobin release. The remaining parasites were incubated with either D-sorbitol (300 mM), L-alanine (300 mM), L-glutamine (300 mM) or choline chloride (160 mM) at 37 C. Aliquots of cell suspensions were taken at 0, 5, 10, 15, 30, or 60 min. Cells and debris were pellet for 30 s at 10,000 × *g* and supernatant was transferred to 96 Well flat bottom assay plates. Absorbance at 540 nm was measured using a BioTek Synergy Neo2 plate reader. Hemoglobin release is given as fraction of maximum release (100%, defined as the release from treatment with 1% Triton X-100).

## Supplementary Material

evag203_Supplementary_Data

## Data Availability

Dryad: https://doi.org/10.5061/dryad.cfxpnvxmw.
